# Exploring the Influence of Left Dorsolateral Prefrontal Cortex Intermittent Theta‐Burst Stimulation on Working Memory Load: An EEG Study in Healthy Participants

**DOI:** 10.1111/cns.70486

**Published:** 2025-06-20

**Authors:** Ya‐wen Li, Qian Ding, Jing Chen, Yu‐hong Huang, Shan‐tong Yao, Zheng‐hong Chen, Yue Lan, Guang‐qing Xu

**Affiliations:** ^1^ Guangdong Cardiovascular Institute Guangdong Provincial People's Hospital, Guangdong Academy of Medical Sciences Guangzhou China; ^2^ Department of Rehabilitation Medicine Guangdong Provincial People's Hospital (Guangdong Academy of Medical Sciences), Southern Medical University Guangzhou China; ^3^ Department of Rehabilitation Medicine The First Affiliated Hospital, Sun Yat‐Sen University Guangzhou China; ^4^ Department of Rehabilitation Medicine Guangzhou First People's Hospital, School of Medicine, South China University of Technology Guangzhou China

**Keywords:** EEG, functional connectivity, intermittent theta‐burst stimulation, N‐back, working memory capacity

## Abstract

**Aims:**

Working memory (WM), a short‐term cognitive process involving the prefrontal cortex, is critical for daily functioning. This study investigated the effects of iTBS on WM performance and its neural mechanisms in healthy adults.

**Methods:**

Thirty‐one healthy adults completed 1‐back and 2‐back tasks while undergoing EEG recording. Each received both active and sham iTBS over the left dorsolateral prefrontal cortex separately. Behavioral performance, EEG power, and functional connectivity were analyzed.

**Results:**

Active iTBS significantly increased theta power in prefrontal cortices during the 1‐back task. It also enhanced alpha band connectivity between the left prefrontal and right parietal cortices. In the 2‐back task, iTBS increased beta band connectivity between the right prefrontal and right parietal cortices, and alpha band connectivity between the left and right parietal cortices. No significant behavioral differences were found.

**Conclusion:**

These findings suggest that iTBS effects on WM are primarily reflected in large‐scale oscillatory network dynamics, rather than solely in localized cortical activity or behavioral performance.

## Graphical abstract

This study explores the effects of intermittent theta burst stimulation (iTBS) on working memory in healthy adults. iTBS modulated neural oscillatory networks, enhancing theta and alpha connectivity between prefrontal and parietal cortices, but no significant behavioral differences were observed.

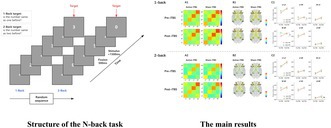

## 1 Introduction

Working memory (WM) refers to the cognitive process of temporarily storing and manipulating information, serving as a fundamental component of daily activities and complex cognitive tasks. It encompasses multiple cognitive processes, including information updating, retention, manipulation, and interference suppression, and it serves as the foundation for higher‐order cognitive functions, such as executive control, learning, decision‐making, and problem‐solving [1, 2]. With the advancement of our understanding of brain mechanisms, studies have shown that working memory relies on the interaction of multiple brain regions, particularly the prefrontal cortex (PFC) and the parietal cortex (PC) [3, 4]. Among these, the dorsolateral prefrontal cortex (DLPFC) plays a crucial role in the updating, control regulation, and load processing of WM [5, 6].

The role of EEG activity in WM has been extensively validated, especially the involvement of different EEG frequency bands, which are closely linked to various cognitive processes of WM [7, 8]. The theta band is typically associated with information maintenance and updating [9, 10]. Alpha rhythms are primarily related to attentional inhibition and information filtering [11, 12]. Beta activity is essential for the stability of cognitive control and information processing [13]. Additionally, the functional connectivity (FC) between brain regions, particularly the coordinated interaction between the prefrontal and parietal regions, is critical for WM performance [14]. Recent studies using functional near‐infrared spectroscopy (fNIRS) have demonstrated that reduced brain network efficiency, coupled with compensatory over‐activation in the prefrontal cortex, may contribute to poorer cognitive performance during complex dual tasks. These findings highlight the importance of examining functional connectivity changes across multiple brain regions to better understand the neural mechanisms underlying cognitive control and working memory [15].

In recent years, transcranial magnetic stimulation (TMS) has gained widespread use in cognitive neuroscience research and clinical intervention as a non‐invasive brain stimulation technique. Specifically, repetitive transcranial magnetic stimulation (rTMS) and intermittent theta burst stimulation (iTBS) have been shown to modulate brain activity and influence cognitive function [16]. Compared to rTMS, iTBS has demonstrated a more robust and lasting effect in enhancing cortical excitability [17]. Research has shown that high‐frequency repetitive TMS of the left DLPFC in healthy individuals can significantly improve WM performance in tasks, such as the language digit span, and the visual–spatial 2‐back task [18]. However, some studies have reported that stimulating the left DLPFC does not yield a positive effect on WM performance. In contrast, stimulation of the right DLPFC and bilateral posterior parietal cortex (PPC) appears to have a more pronounced effect on WM [19]. Furthermore, the efficacy and safety of iTBS have been validated in patients with post‐stroke cognitive impairment (PSCI) [20]. In Alzheimer's disease (AD), DLPFC plasticity is impaired, and enhancing the function of this region may offer a potential intervention target for improving WM [21].

In addition, sex differences have been reported to influence cognitive functions, including WM performance. Behavioral studies suggest that females tend to perform better on verbal WM tasks, whereas males may show advantages in spatial WM [22]. Neuroimaging findings further reveal sex‐specific activation patterns during WM engagement, which may reflect distinct cognitive strategies or neural efficiencies [23]. Therefore, when evaluating the effects of neuromodulation interventions like iTBS, it is important to consider potential sex‐related variability.

Despite these findings, whether iTBS targeting the left DLPFC can significantly enhance WM and its underlying neural mechanisms remains unclear. The present study aimed to investigate the effects of iTBS applied to the left DLPFC on EEG activity and functional connectivity during a visual n‐back task in healthy adults. We focused on oscillatory power and interregional synchronization across the left and right PFC and PPC under two levels of WM load (1‐back and 2‐back). Moreover, we included sex as a between‐subject factor to explore its moderating role. We hypothesized that, compared to sham stimulation, active iTBS would enhance behavioral performance and increase local activity and frontoparietal connectivity across the regions of interest.

## 2 Materials and Methods

### 2.1 Participants

A total of 31 right‐handed, healthy adult participants were recruited for this study, consisting of 17 females and 14 males, with an average age of 22.3 years (range = 20–24, SD = 0.991). Prior to participation, all participants provided written informed consent and completed a TMS screening questionnaire. Inclusion criteria required participants to have normal or corrected‐to‐normal vision and no history of neurological or psychiatric disorders. The study was approved by the Ethics Review Committee of Guangdong Provincial People's Hospital (KY2023‐1079‐02) and has been registered at the Chinese Clinical Trial Registry (ChiCTR2500097678). This study adhered to the ethical principles of the Declaration of Helsinki.

### 2.2 Experimental Design

This study employed a within‐subjects, randomized, single‐blind design. Participants completed the experiment on two separate days, with a minimum 1‐week interval between sessions [24]. On each day, they received either active iTBS or sham stimulation. Each session consisted of baseline 1‐ and 2‐back tasks, followed by either active or sham iTBS stimulation, and a subsequent post‐stimulation 1‐ and 2‐back task. EEG data were continuously recorded throughout the experiment.

### 2.3 iTBS Stimulation

iTBS was applied to the left DLPFC using an NS5000 magnetic stimulator (Erid Medical Co. Ltd., Wuhan, China) equipped with a 70 mm figure‐8 coil (biphasic pulse, pulse width = 350 ms). The protocol consisted of three 50‐Hz pulses, repeated at 5 Hz, with a 2‐s burst train repeated every 10 s for a total of 192 s (600 pulses in total) [17, 25]. The stimulation intensity was set to 80% of the participant's active motor threshold (AMT) [25, 26], which has been demonstrated to be safe and effective [27]. Before the iTBS application, the resting motor threshold (RMT) was measured by applying TMS to the right motor cortex and recording electromyography activity from the first dorsal interosseous muscle (FDI). Participants were seated comfortably with their eyes open throughout the measurement [28, 29]. The TMS coil was angled 45° to the midline tangent, and the participant's arms were relaxed to locate the optimal scalp position for eliciting the maximal motor‐evoked potential (MEP) in the FDI [29, 30]. The RMT was defined as the minimum stimulation intensity required to evoke at least five MEPs exceeding 50 μV in ten trials [31]. A neuronavigation system (Visor2, ANT Neuro, The Netherlands) was used to ensure precise coil positioning at the motor hotspot [29].

iTBS was applied to the left DLPFC, and the positioning method was based on the position of the F3 electrode, using the international 10–20 system for calibration [32]. Participants remained still during iTBS stimulation. For active stimulation, the TMS coil was placed tangentially to the scalp over the left DLPFC, with the handle angled approximately 45° posterior‐laterally to the midline, consistent with the orientation used during motor threshold localization. Participants wore earplugs throughout both active and sham sessions to reduce coil noise and minimize auditory cues. Sham stimulation was administered using the same coil, placed perpendicularly to the scalp with the same stimulation intensity (80% AMT), producing similar auditory and somatosensory sensations but without effective cortical stimulation [33]. Participants were blind to the type of stimulation to minimize placebo effects, and no participant could distinguish between real and sham stimulation.

### 2.4 N‐Back Task

**FIGURE 1:**
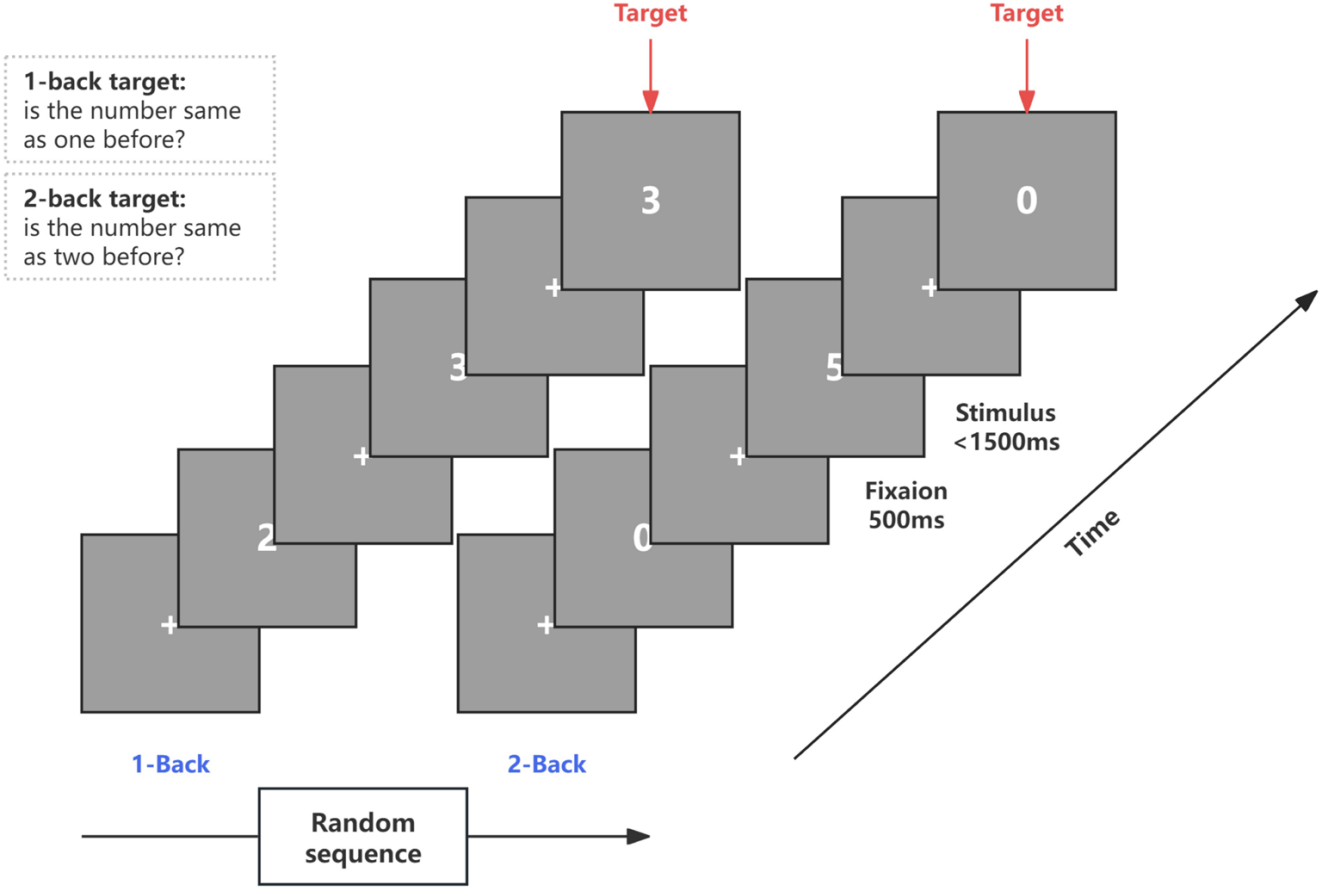
Structure of the N‐back task. Participants performed a randomized sequence of digits under two cognitive load conditions: 1‐back and 2‐back. In the 1‐back condition, a target was defined as a digit matching the one presented immediately before. In the 2‐back condition, a target was a digit that matched the one presented two trials earlier. Each trial began with a fixation cross displayed for 500 ms, followed by a digit presented for up to 1500 ms. Participants were required to respond as quickly and accurately as possible to indicate whether the current digit was a target or non‐target. Red arrows indicate example target trials.

The N‐back task assessed WM capacity under two load conditions: 1‐back (low memory load) and 2‐back (high memory load). The task structure is outlined in Figure 1. Each trial presented a digit (0–9) on the screen. Participants were required to respond by pressing the N or M key: M if the current stimulus was the target (matching the stimulus presented 1 or 2 trials ago), and N if it was not. Each condition comprised 90 stimuli, with 50% being target stimuli. In the 1‐back condition, the target number was the same as the immediately preceding digit; in the 2‐back condition, the target number matched the digit presented two trials earlier.

Each stimulus was displayed on the screen for a maximum of 1500 milliseconds, and participants were instructed to respond within this time frame. A 500‐ms interstimulus interval separated consecutive stimuli, during which a fixation cross was displayed. Before the formal task, participants practiced the 1‐back condition followed by the 2‐back condition, with 30 practice trials for each [34]. After completing the practice, the screen displayed the message “Practice is over. You will now begin the formal test.” The experiment utilized a block design consisting of 6 blocks: 3 for the 1‐back task and 3 for the 2‐back task, with block order randomized. Each block contained 30 trials. All stimuli were presented using E‐Prime 3.0 software (Psychology Software Tools Inc., Pittsburgh, PA, USA) on a 14‐inch Lenovo computer monitor.

### 2.5 Electroencephalography (EEG)

#### 2.5.1 EEG Recording and Preprocessing

EEG data were recorded during the experiment while participants sat comfortably in a quiet, dimly lit room. EEG signals were recorded using a TMS‐compatible EEG cap (ANT Neuro, Enschede, Netherlands) with 64 Ag/AgCl electrodes arranged according to the international 10–20 system [35]. CPz was used as the online reference, and signals were amplified using an eego amplifier (ANT Neuro, Enschede, Netherlands). Electrode impedance was maintained below 10 kΩ, and data were sampled at 2048 Hz.

Offline analysis was conducted in MATLAB 2022b (MathWorks Inc., Natick, USA) using custom scripts. Preprocessing was performed using the EEGLAB toolbox (version 14.1.2b) [36]. The data were bandpass filtered (0.1–30 Hz), and a notch filter at 50 Hz was applied to remove power line interference. The sampling rate was reduced to 1000 Hz, and data segments from −200 to 800 ms relative to stimulus onset were extracted, with baseline correction based on the −200 ms pre‐stimulus period. Only correct trials were included in the analysis [37]. Each segment was visually inspected, and artifact‐containing segments were discarded. Independent component analysis (ICA) was applied to identify and remove typical non‐neural artifacts, including components related to eye blinks, horizontal eye movements, muscle activity, and electrocardiographic (ECG) artifacts. These components were identified based on their characteristic spatial topographies, time courses, and frequency spectra, and were removed before further analysis [38]. Trials with amplitudes exceeding 100 μV were excluded, and the data were re‐referenced using the average signal of all scalp electrodes.

#### 2.5.2 EEG Analysis

Time‐frequency analysis (TFA) was performed using a custom MATLAB script. The Morlet wavelet transform was applied to compute the time‐frequency representation of the EEG data [39], covering frequencies from 3.9 to 30 Hz, including theta (4–7 Hz), alpha (8–13 Hz), and beta (14–30 Hz) bands. The power changes in each frequency band were calculated and presented as time‐frequency plots. Each trial was baseline normalized using a pre‐stimulus window (−200 to −50 ms) and converted to decibel (dB) units [40]. The ROIs included the left PFC (AF3, F1, F3, F5), right PFC (AF4, F2, F4, F6), left PPC (P3, P5, P7, CP1), and right PPC (P4, P6, P8, CP2), with average power calculated for each region.

FC analysis was performed to assess the synchrony between different brain regions using the phase‐locking value (PLV). Unlike previous studies that primarily employed amplitude‐based or correlation‐based measures to characterize dynamic functional connectivity [41, 42], the present study adopted the PLV, which specifically captures phase synchrony between neural signals. PLV is less affected by signal amplitude fluctuations or volume conduction and provides higher sensitivity to short‐lived, transient changes in inter‐regional coupling, making it particularly suitable for detecting subtle neuromodulatory effects of iTBS. The PLV was calculated between pairs of ROIs: left prefrontal–left posterior parietal (LF–LP), left prefrontal–right parietal (LF–RP), right prefrontal–left posterior parietal (RF–LP), right prefrontal–right posterior parietal (RF–RP), left prefrontal–right prefrontal (LF–RF), and left posterior parietal–right posterior parietal (LP–RP). PLV values, ranging from 0 (no synchrony) to 1 (complete synchrony), were computed using the Morlet wavelet transform and fast Fourier transform (FFT). The average PLV for each pair of regions was calculated and visualized using the BrainNet Viewer toolbox in MATLAB.

### 2.6 Statistical Analysis

All statistical analyses were performed using SPSS 26 (IBM, USA). Data were analyzed using a 2 (stimulation: active vs. sham) × 2 (session: pre‐iTBS vs. post‐iTBS) repeated measures analysis of variance (ANOVA). Data were found to meet the normality assumption using the Shapiro–Wilk test. To control for multiple comparisons, *p*‐values were adjusted using the Bonferroni correction method. If the main effect or interaction effect was significant, we would perform post hoc simple effect analysis to further explore the specific differences between the conditions. For all analyses, the statistical significance was set at *p* < 0.05, and adjusted *p*‐values are presented. Gender was included as a between‐subjects factor in the mixed‐design ANOVA to explore its potential moderating effects on behavioral and EEG outcomes following iTBS.

## 3 Result

### 3.1 Behavioral Results (Reaction Time and Accuracy)

**FIGURE 2:**
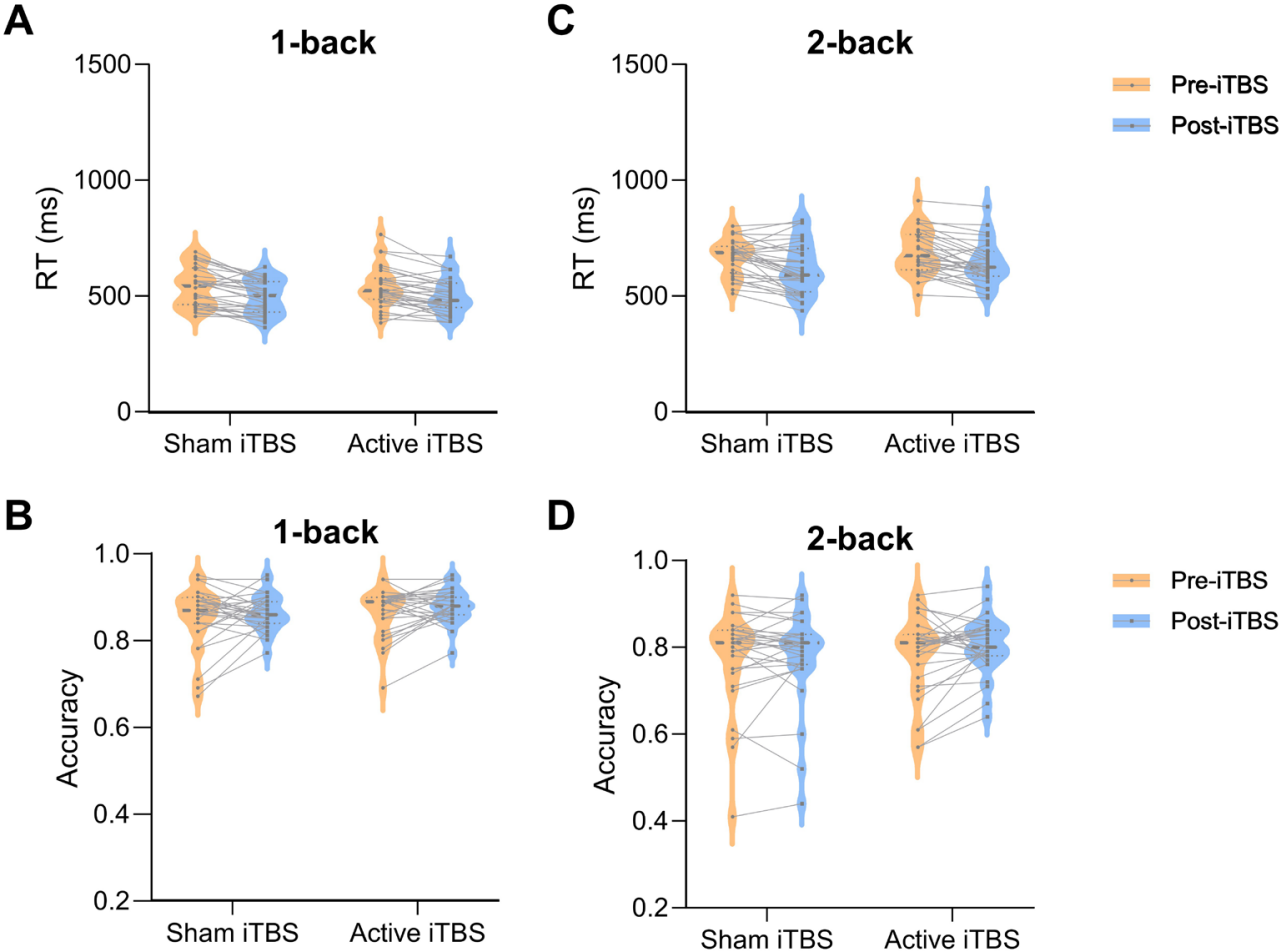
Behavioral results. (A, B) Accuracy results demonstrate no significant effects in both the 1‐back (A) and 2‐back (B) conditions. (C, D) Reaction time results show no significant effects in both the 1‐back (C) and 2‐back (D) conditions. The violin plots depict the distribution of individual data points, where wider sections indicate higher data density. The thick central dashed line represents the median, whereas the thin dashed lines denote the interquartile range. Overlaid lines connect individual participants' data across sessions, highlighting within‐subject changes.

Figure 2 presents the behavioral results. In the 1‐back condition, neither stimulation nor session had a significant effect on accuracy. Neither stimulation nor session had a significant effect in the 1‐back or 2‐back conditions.

In the 1‐back condition, Stimulation had no significant effect on reaction time. However, the session showed a significant main effect [*F*_(1,30)_ = 39.629, *p <* 0.001, *η* ^2^ = 0.559], indicating a significant decrease in reaction time across sessions. No interaction effect was found between stimulation and session [*F*_(1,30)_ = 0.002, *p* = 0.969, *η* ^2^ = 0.569].

In the 2‐back condition, the main effect of stimulation on reaction time was also non‐significant. The session showed a significant main effect on reaction time, [*F*_(1,30)_ = 54.186, *p* < 0.001, *η* ^2^ = 0.644].

### 3.2 EEG Post‐Stimulation Power

**FIGURE 3:**
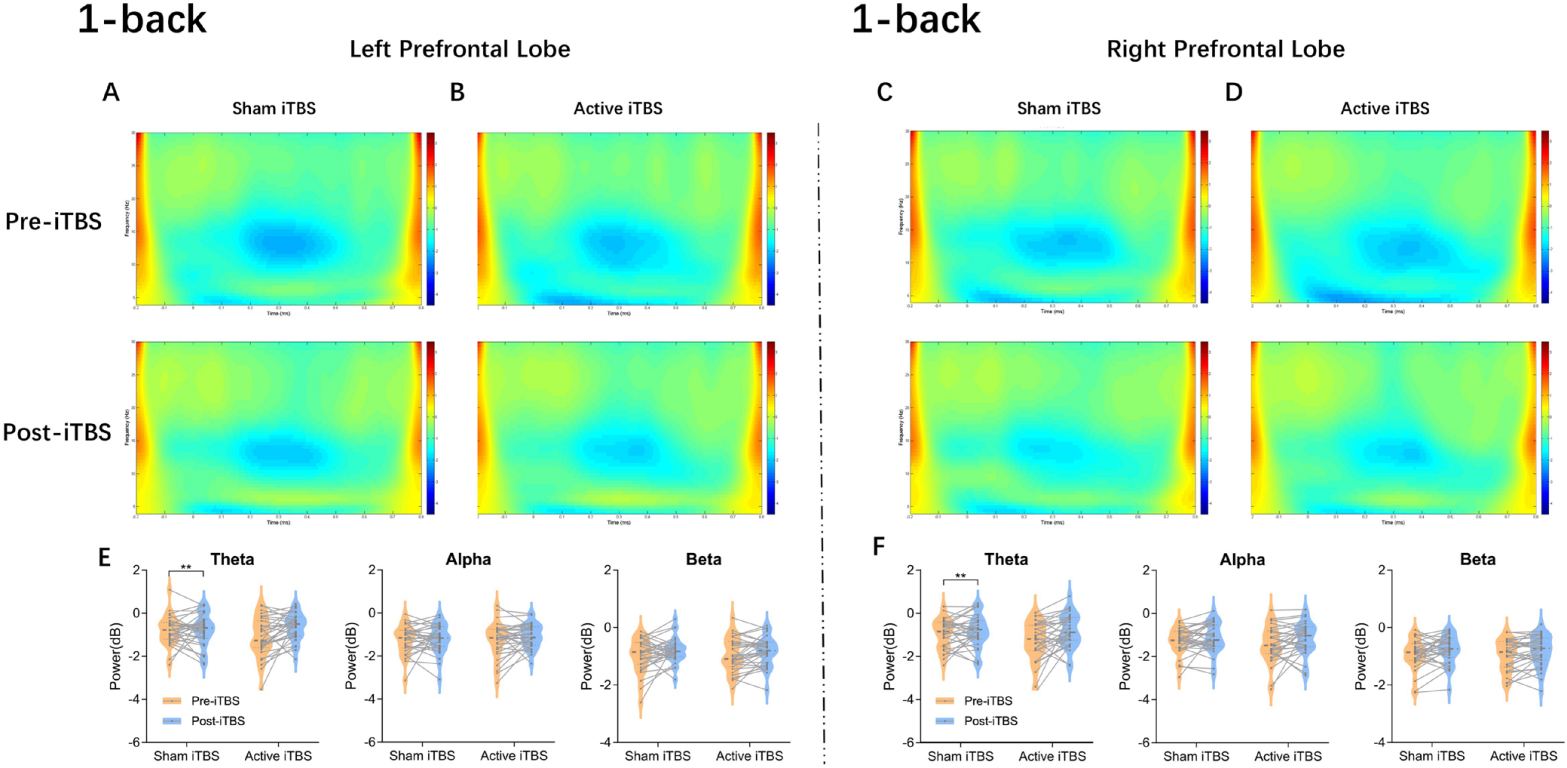
Post‐stimulation power in the left and right PFCs during the 1‐back condition. Time‐frequency representations of left prefrontal power changes for sham (A) and active iTBS (B) conditions, before (top) and after (bottom) stimulation. Right prefrontal power changes for sham (C) and active iTBS (D) conditions. Violin plots of theta, alpha, and beta power distributions for the left (E) and right (F) PFC. The thick central dashed line represents the median, whereas the thin dashed lines denote the interquartile range. The color bar of the TF plots represents the value of EEG power on the dB scale. **p < 0.01.

Figure 3 presents the post‐stimulation power results for the left and right prefrontal cortices (PFCs) in the 1‐back condition.

In the left PFC, the main effect of stimulation on theta band activity was not significant. However, a significant main effect of session was observed [*F*_(1,30)_ = 4.893, *p* = 0.035, *η* ^2^ = 0.140], along with a significant interaction between stimulation and session [*F*_(1,30)_ = 5.060, *p* = 0.032, *η* ^2^ = 0.144]. Simple effects analysis revealed that theta power significantly increased from pre‐ to post‐stimulation in the active stimulation condition (*p* = 0.005), but not in the sham condition (*p* = 0.756). No significant effects were found for the alpha and beta frequency bands.

Similarly, in the right PFC, although the main effect of stimulation on theta band power was not significant, a significant main effect of session was found [*F*_(1,30)_ = 6.289, *p* = 0.018, *η* ^2^ = 0.173], along with a significant interaction between stimulation and session [*F*_(1,30)_ = 5.123, *p* = 0.031, *η* ^2^ = 0.146]. Further analysis indicated a significant increase in theta band power before and after stimulation in the active stimulation condition (*p* = 0.006), whereas no significant change was observed in the sham stimulation condition (*p* = 0.350). No significant effects in alpha or beta bands.

In contrast, no significant changes were detected in any frequency band within the left and right posterior parietal cortices (PPCs) in the 1‐back condition.

In the 2‐back condition, no significant power alterations in any frequency band in either the left or right PFC and PPC, regardless of whether active or sham iTBS stimulation was applied.

### 3.3 EEG Functional Connectivity

#### 3.3.1 Theta Band

**FIGURE 4:**
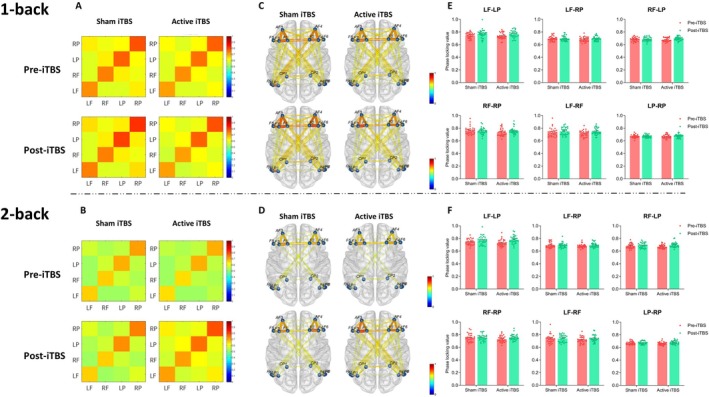
Theta band functional connectivity. (A, B) represent the brain connectivity matrices between the left and right prefrontal and parietal cortices under the 1‐back (A) and 2‐back (B) conditions, respectively. (C, D) illustrate the brain connectivity networks for each group under the 1‐back (C) and 2‐back (D) conditions. The column plots (E, F) depict the phase‐locking value of different brain regions under the 1‐back (E) and 2‐back (F) conditions, with individual data points overlaid. Error bars represent the average standard error. The color bars in the connectivity matrices and brain connectivity networks indicate the synchronization strength, ranging from 0 to 1, with higher values representing stronger synchronization. LF, Left prefrontal; LP, Left posterior parietal; RF, Right prefrontal; RP, Right posterior parietal.

Figure 4 shows the FC results for the theta band. In the 1‐back condition, stimulation did not significantly affect FC in any region. Session had a significant main effect on FC in LF_LP [*F*_(1,30)_ = 13.544, *p =* 0.001, *η* ^2^ = 0.311], and LF_RF [*F*_(1,30)_ = 7.694, *p =* 0.009, *η* ^2^ = 0.204]. There were no significant interaction effects between stimulation and session in any of the regions.

In the 2‐back condition, the session had a significant main effect on FC in LF_LP [*F*_(1,30)_ = 22.779, *p* < 0.001, *η* ^2^ = 0.432], and LP_RP [*F*_(1,30)_ = 6.942, *p =* 0.013, *η* ^2^ = 0.188]. No significant effects were found in other regions.

#### 3.3.2 Alpha Band

**FIGURE 5:**
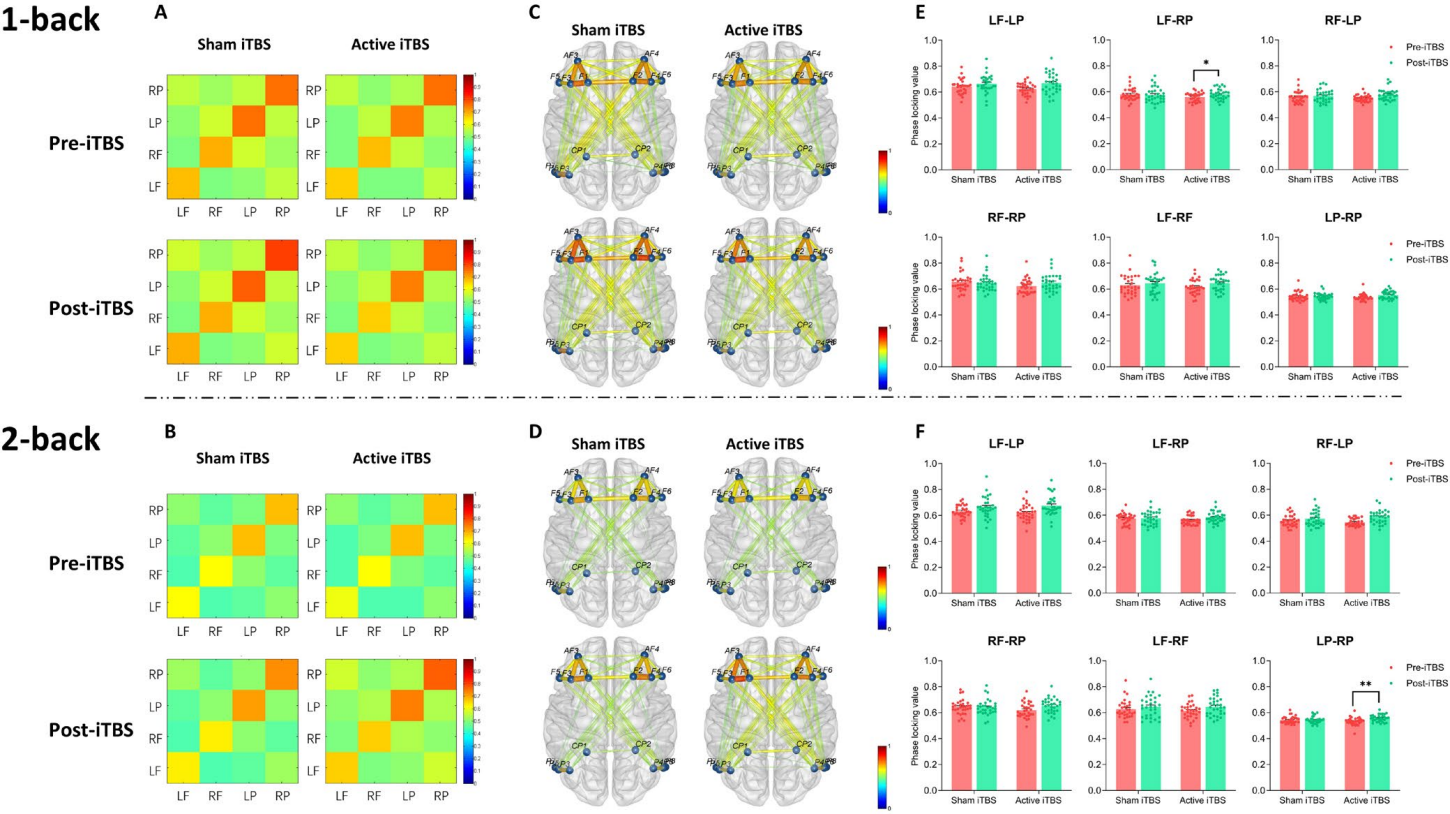
Alpha band functional connectivity. (A, B) represent the brain connectivity matrices between the left and right prefrontal and parietal cortices under the 1‐back (A) and 2‐back (B) conditions, respectively. (C, D) illustrate the brain connectivity networks for each group under the 1‐back (C) and 2‐back (D) conditions. The column plots (E, F) depict the phase‐locking value of different brain regions under the 1‐back (E) and 2‐back (F) conditions, with individual data points overlaid. Error bars represent the average standard error. The color bars in the connectivity matrices and brain connectivity networks indicate the synchronization strength, ranging from 0 to 1, with higher values representing stronger synchronization. LF, Left prefrontal; LP, Left posterior parietal; RF, Right prefrontal; RP, Right posterior parietal. *p < 0.05, **p < 0.01.

Figure 5 shows the FC results for the alpha band.

In the 1‐back condition, stimulation did not show a significant effect on FC in LF_LP, RF_LP, or LF_RF. Session had a significant main effect on FC in these regions: LF_LP [*F*_(1,30)_ = 18.302, *p <* 0.001, *η* ^2^ = 0.379], RF_LP [*F*_(1,30)_ = 12.323, *p =* 0.001, *η* ^2^ = 0.291], and LF_RF [*F*_(1,30)_ = 4.213, *p =* 0.049, *η* ^2^ = 0.127]. *F*or LF_RP, the interaction between stimulation and session was significant [*F*_(1,30)_ = 4.431, *p =* 0.044, *η* ^2^ = 0.129]. Simple effect analysis revealed that, under active stimulation, there was a significant difference in FC before and after stimulation (*p =* 0.015), whereas no significant difference was found under sham stimulation (*p =* 0.624). The interaction effect was also significant for RF_RP [*F*_(1,30)_ = 5.027, *p =* 0.033, *η* ^2^ = 0.144], with active stimulation showing a near‐significant difference in FC (*p* = 0.054), whereas no difference was found under sham stimulation (*p* = 0.548). For LF_RF, neither stimulation nor session showed a significant effect.

In the 2‐back condition, stimulation did not affect FC in LF_LP, RF_LP, or LP_RP. Session had a significant main effect on these regions: LF_LP [*F*_(1,30)_ = 15.621, *p <* 0.001, *η* ^2^ = 0.342], RF_LP [*F*_(1,30)_ = 17.952, *p <* 0.001, *η* ^2^ = 0.374], and LP_RP [*F*_(1,30)_ = 6.748, *p =* 0.014, *η* ^2^ = 0.184]. For LF_RP and RF_RP, neither stimulation nor session had a significant effect. For LF_RF, whereas stimulation did not show a main significant effect, there was a significant interaction effect between stimulation and session [*F*_(1,30)_ = 9.505, *p =* 0.004, *η* ^2^ = 0.241]. Simple effect analysis showed that under active stimulation, FC before and after stimulation was significantly different (*p =* 0.001), whereas no difference was observed under sham stimulation (*p =* 0.977).

#### 3.3.3 Beta Band

**FIGURE 6:**
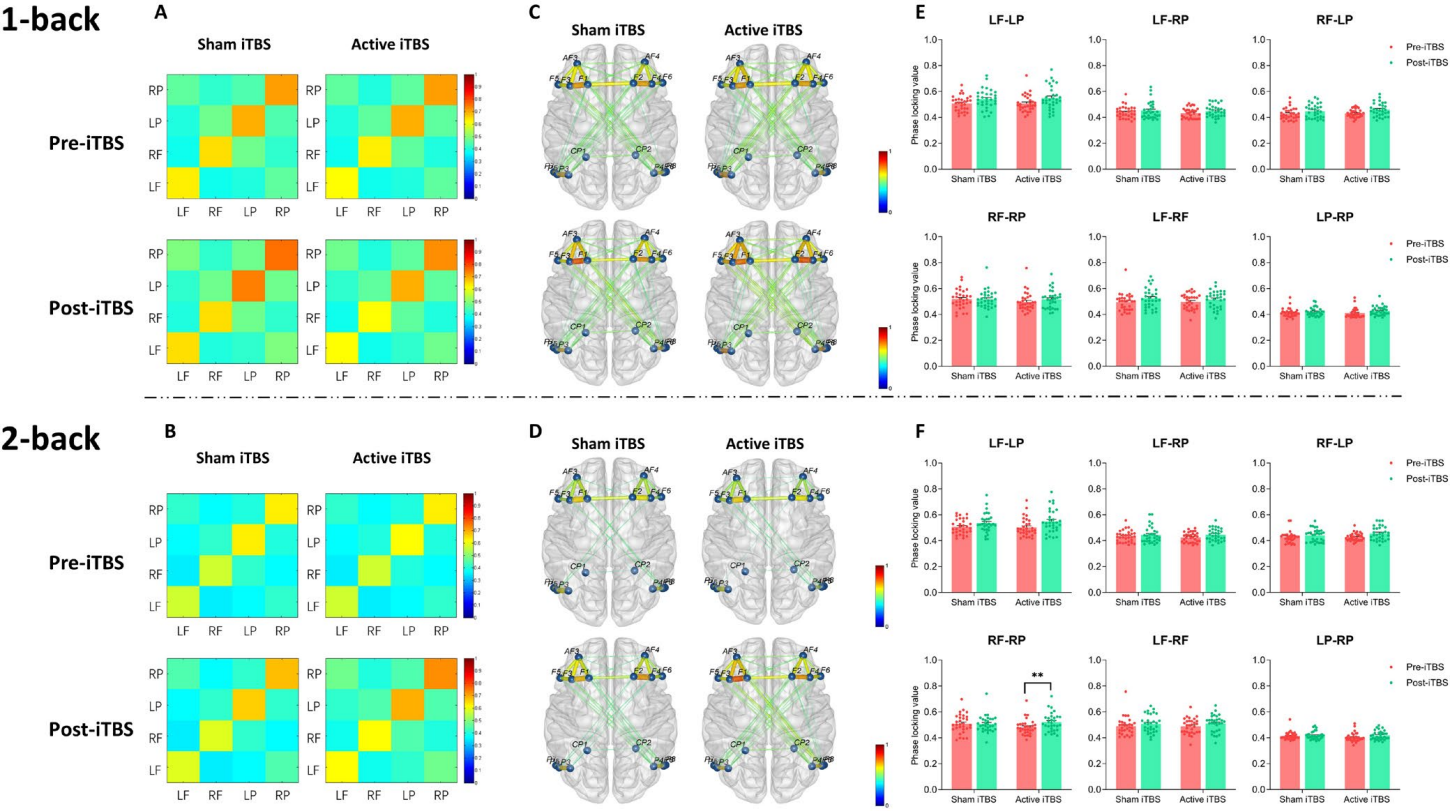
Beta band functional connectivity. (A, B) represent the brain connectivity matrices between the left and right prefrontal and parietal cortices under the 1‐back (A) and 2‐back (B) conditions, respectively. (C, D) illustrate the brain connectivity networks for each group under the 1‐back (C) and 2‐back (D) conditions. The column plots (E, F) depict the phase‐locking value of different brain regions under the 1‐back (E) and 2‐back (F) conditions, with individual data points overlaid. Error bars represent the average standard error. The color bars in the connectivity matrices and brain connectivity networks indicate the synchronization strength, ranging from 0 to 1, with higher values representing stronger synchronization. LF, Left prefrontal; LP, Left posterior parietal; RF, Right prefrontal; RP, Right posterior parietal. *p < 0.05.

Figure 6 presents the FC results for the beta band.

In the 1‐back condition, stimulation had no significant effect on FC in RF_LP and LP_RP. Session had a significant effect on the FC of RF_LP [*F*_(1,30)_ = 14.164, *p =* 0.001, *η* ^2^ = 0.321], and LF_RF [*F*_(1,30)_ = 4.199, *p =* 0.049, *η* ^2^ = 0.123]. No significant main or interaction effects were found for the FC of LF_LP, LF_RP, RF_RP, and LF_RF.

In the 2‐back condition, session had a significant effect on FC in LF_LP [*F*_(1,30)_ = 8.806, *p =* 0.006, *η* ^2^ = 0.227], and RF_LP [*F*_(1,30)_ = 12.801, *p =* 0.001, *η* ^2^ = 0.299]. There was a significant interaction effect between stimulation and session [*F*_(1,30)_ = 6.687, *p =* 0.015, *η* ^2^ = 0.182]. Simple effect analysis showed that under active stimulation, FC before and after stimulation was significantly different (*p =* 0.002), whereas no difference was observed under sham stimulation (*p =* 0.866).

### 3.4 Effects of Sex

Sex was included as a between‐subjects factor in all 2 (stimulation: active vs. sham) × 2 (session: pre‐ vs. post‐iTBS) repeated‐measures ANOVAs for behavioral (accuracy, reaction time) and EEG (power, FC) outcomes. No significant main effects of sex or sex × stimulation/session interactions were observed for any measure (all *p* > 0.05), indicating that the effects of iTBS did not differ between males and females in this sample.

## 4 Discussion

### 4.1 Dissociation Between EEG and Behavioral Outcomes

This study investigated the effects of iTBS on behavioral performance and EEG activity during N‐back WM tasks under two cognitive load levels. Although no significant behavioral changes were observed, iTBS significantly enhanced EEG FC, particularly in the alpha and beta bands, and increased theta power during low‐load conditions.

Although significant modulations were observed in EEG‐derived measures following stimulation, behavioral performance did not exhibit parallel changes. This divergence may reflect the higher sensitivity of electrophysiological markers in detecting transient or subthreshold neural adaptations that are not yet sufficient to drive measurable improvements in task performance. Particularly in young, cognitively healthy individuals, behavioral outcomes on relatively low‐demand tasks may approach ceiling effects, limiting observable gains. Moreover, we intentionally restricted our sample to healthy young adults to minimize interindividual variability and confounding age‐related factors in cognitive and neural responses. Although this enhances internal validity, it may limit generalizability. Future studies should consider age‐diverse populations to explore the broader applicability of iTBS effects. Furthermore, iTBS‐induced plasticity may manifest initially at the neural level, necessitating longer periods of consolidation or the engagement of more challenging cognitive demands to translate into behavioral benefits.

### 4.2 Task‐Load‐Dependent Theta Modulation

In the power analysis, for the 1‐back task, we observed a significant increase in theta band activity in both the left and right frontal cortices after iTBS stimulation. Theta waves (4–8 Hz) are generally associated with processes, such as information encoding, maintenance, and updating in WM [43]. In the low‐load 1‐back task, theta power significantly increased in both the right and left prefrontal cortex, which may reflect the role of these regions in information processing and interference suppression. Previous studies using neuromagnetic imaging have shown that theta activity in the frontal cortex increases as memory load increases [44].

In contrast, we did not observe a similar increase in the 2‐back task. This dissociation may be explained by differences in cognitive load and the allocation of neural resources. Frontal theta activity has been consistently linked to working memory maintenance and cognitive control, particularly under moderate cognitive demands [45]. The relatively low task load in the 1‐back condition may allow stimulation‐induced modulation to manifest more clearly in the theta band, as sufficient neural capacity remains available. In contrast, the higher demands of the 2‐back task may saturate working memory systems, limiting the observable effects of iTBS on theta oscillations [46]. Furthermore, prior studies have indicated that under increased cognitive load, task‐related processing may reduce reliance on frontal theta synchronization [47]. It has also been suggested that theta enhancement reflects an optimized, rather than maximal, level of cognitive engagement [48]. Therefore, in the 2‐back condition, either compensatory mechanisms are activated, or longer‐term or more intensive stimulation may be needed for measurable neural modulation.

### 4.3 Alpha and Beta Connectivity Changes Under Cognitive Load

In the FC analysis, we found that iTBS targeting the left DLPFC enhanced alpha band synchronization between the left PFC and the right PPC during the 1‐back task. This suggests improved functional coupling under low cognitive load, likely reflecting more efficient top‐down modulation of attention and working memory maintenance. In contrast, during the more demanding 2‐back task, increased alpha band phase synchronization was observed between the left and right PPC, indicating that iTBS may facilitate compensatory interhemispheric communication within parietal regions when task demands exceed the capacity of prefrontal systems [48]. Increased alpha band phase synchronization is thought to reflect not only the inhibition of task‐irrelevant networks but also the functional integration of task‐relevant systems [49]. The observed increase in alpha synchronization between bilateral PFC regions following stimulation may indicate enhanced coordination within the frontoparietal control network, possibly supporting higher‐order executive functions [50].

In the 2‐back task, we also observed enhanced FC between the right PFC and right PPC in the beta frequency band. Beta waves are commonly associated with task execution and cognitive control [51, 52]. By enhancing collaboration between the PFC and other cognitive regions (such as the PPC), iTBS may optimize cognitive resource allocation and control, allowing participants to make more effective decisions, plan, and execute tasks during high‐load WM tasks.

These results highlight that iTBS over the left DLPFC does not exert uniform effects across task loads; rather, its neuromodulatory impact appears load‐dependent, enhancing frontal–parietal integration during low‐demand tasks while promoting parietal compensatory mechanisms under higher demands. This aligns with the role of the left DLPFC in adaptive cognitive control and its capacity to flexibly modulate large‐scale networks in response to varying task requirements [53].

### 4.4 Implications for Clinical Applications

Taken together, our findings provide insights into the neuromodulatory mechanisms of iTBS and highlight its task load‐dependent effects on neural oscillations and functional connectivity. Beyond healthy populations, WM deficits are prevalent in aging, mild cognitive impairment, AD, and stroke patients [54]. Studies indicate that dysregulated low‐ and high‐frequency oscillatory interactions contribute to memory decline in these populations [55, 56]. In subacute stroke, beta oscillations have been strongly associated with WM function and prognosis [57]. These findings highlight the potential of non‐invasive brain stimulation as a therapeutic tool for WM enhancement, particularly by modulating oscillatory patterns to optimize cognitive function. Although no adverse event was reported in the current study, potential side effects of iTBS, such as transient headache or scalp discomfort, should be acknowledged. These risks are typically minimal in healthy individuals but warrant attention in clinical applications.

## 5 Limitations

Although this study provides new insights into the regulatory effects of iTBS on WM, there are some limitations. First, we investigated only a single iTBS session and a single type of cognitive task. Although our findings provide preliminary evidence of the neuromodulatory effects of iTBS, the effects of a single stimulation session may be limited in scope and duration. Future studies should explore the cumulative effects of repeated iTBS sessions, which may produce more robust and longer‐lasting changes in both neural activity and behavioral outcomes. In addition, the lack of long‐term follow‐up in this study prevents assessment of the durability and stability of iTBS‐induced changes over time. Longitudinal studies are warranted to evaluate whether the observed effects persist or evolve with repeated stimulation or extended post‐stimulation periods. Second, although we examined theta, alpha, and beta frequency bands, future research could investigate higher frequency bands (e.g., gamma) or apply finer spectral resolution to better characterize the frequency‐specific roles of neural oscillations in cognitive processing. Third, the sample size in this study was relatively small and consisted solely of healthy young adults. To enhance generalizability, future research should recruit larger and more diverse samples, including individuals across different age groups and clinical populations, to validate the efficacy and applicability of iTBS across broader demographics.

## 6 Conclusion

In summary, this study suggests that the effects of iTBS intervention on the left DLPFC in healthy participants appear to be mediated by enhancing related neurophysiological processes. This provides a new theoretical foundation for the potential application of iTBS in cognitive interventions and offers important implications for future research and clinical applications.

## Author Contributions

**Ya‐wen Li:** writing – original draft, writing – review and editing, visualization, software, methodology, investigation, formal analysis, data curation. **Qian Ding:** writing – review and editing, methodology, investigation, funding acquisition, formal analysis, data curation. **Jing Chen:** writing – review and editing, methodology, investigation. **Yu‐hong Huang:** methodology, investigation. **Shan‐tong Yao:** methodology, investigation. **Zheng‐hong Chen:** supervision, project administration, conceptualization. **Yue Lan:** supervision, resources, project administration, funding acquisition, conceptualization. **Guang‐qing Xu:** writing – review and editing, resources, project administration, funding acquisition, conceptualization.

## Conflicts of Interest

The authors declare no conflicts of interest.

## Data Availability

The data that support the findings of this study are available from the corresponding author upon reasonable request.
